# Evaluating the Feasibility of Using Historical Placebo Control in Osteoarthritis Trials

**DOI:** 10.3390/epidemiologia7010027

**Published:** 2026-02-14

**Authors:** Justine Monseur, Emma Barbeau, Anne-Françoise Donneau, Olivier Bruyère

**Affiliations:** 1 Research Unit Public Health, From Biostatistics to Health Promotion, University of Liège, 4000 Liège, Belgium; jmonseur@uliege.be (J.M.); barbeauemma04@gmail.com (E.B.); afdonneau@uliege.be (A.-F.D.); 2 Research Unit in Public Health, Epidemiology and Health Economics, University of Liège, 4000 Liège, Belgium

**Keywords:** osteoarthritis, placebo, randomized controlled trial

## Abstract

Background/Objectives: Randomized controlled trials (RCTs) are the gold standard for evaluating treatment efficacy, yet recruitment and retention remain challenging, particularly when placebo arms are required. Using historical placebo data may reduce the need for contemporaneous placebo groups, but comparability between historical and real-time placebo responses is uncertain. This study assessed the feasibility of replacing a placebo control group with a historical placebo arm in osteoarthritis (OA) RCTs using several matching approaches. Methods: Data from three published knee OA RCTs (2009, 2013, 2017) were analyzed. The study followed three steps: (1) development of matching techniques using the 2009 and 2013 trials, (2) validation in the 2017 trial, and (3) post hoc analyses comparing placebo responses across trials. Methods included direct covariate adjustment, exact and nearest-neighbor matching, and propensity score matching based on baseline characteristics (age, sex, BMI, OA duration, baseline pain). The main outcome was change in 100 mm visual analogue scale (VAS) pain. Results: Initial attempts showed moderate to good success in adjusting historical placebo response on the VAS using various adjustment methods. However, in the validation process, a significant discrepancy was observed between real placebo VAS changes data and historical placebo VAS changes data, and various matching techniques failed to sufficiently reduce this discrepancy. In the post hoc analysis, despite the application of advanced matching techniques, substantial variability in VAS placebo responses persisted across trials. Even among placebo patients with highly similar baseline characteristics, the VAS changes over time differed significantly between studies. Conclusions: The findings indicate that replacing a real placebo group with a historical placebo in osteoarthritis RCTs is hardly feasible. These results underscore the complexity of placebo effects in osteoarthritis trials and the limitations of historical control data in this context.

## 1. Introduction

Randomized controlled trials (RCTs) are the gold standard for evaluating the effectiveness and safety of experimental interventions. In conventional RCT designs, participants are randomly assigned to an experimental arm or a control arm, which may include a placebo or the current standard of care. The integrity and reliability of RCTs depend on adequate recruitment, retention, and adherence to the assigned interventions. However, recruitment and retention can be challenging, particularly for placebo controls, as patient reluctance to receive an inactive treatment can lead to higher dropout rates or hinder initial enrollment [1,2,3]. These challenges are particularly pronounced in chronic diseases such as osteoarthritis (OA), where placebo-controlled trials require long follow-up periods and significant participant engagement.

To overcome these limitations, an alternative approach is to include historical control (HC) data, which can complement, or in some cases replace, contemporary placebo groups [4]. HC data are derived from previous clinical trials with similar interventions and patient populations, and they provide a potential means of reducing the number of patients required in new trials while maintaining statistical power and methodological robustness [5,6]. The use of HC data may be particularly beneficial in therapeutic areas of high unmet medical need where recruitment of placebo-controlled cohorts may be ethically or logistically challenging [7,8]. In addition, incorporating HC data into trial designs can improve the cost-effectiveness of drug development, streamline trial conduct, and refine treatment effect estimates.

Although HC data offer several advantages, their utilization is accompanied by methodological challenges and inherent limitations [6]. A primary benefit of HC data is their provenance from well-documented clinical trials that adhere to standardized protocols, with clearly defined inclusion and exclusion criteria and meticulous exposure and outcome assessments. Nevertheless, concerns regarding comparability persist, as historical and contemporary study populations may exhibit disparities in demographic composition, disease severity, standard of care, and other contextual factors that influence treatment response [9]. The variability in trial methodologies, including differences in outcome ascertainment, follow-up duration, and censoring criteria, further complicates the direct application of HC data.

A critical issue in using HC data is the potential for selection bias, which arises when systematic differences exist between historical and current patient cohorts [9,10]. Addressing such biases necessitates the employment of advanced statistical methodologies to ensure the comparability of the groups. Several observational research techniques, including restriction, stratification, direct matching, regression-based adjustments, and weighting methods, can be employed to mitigate confounding factors. Propensity score methods, which estimate the probability of assignment to a particular cohort based on observed baseline characteristics, are commonly used to balance treatment and control groups. These techniques can be applied for direct matching, inverse probability weighting, or stratification, thereby improving the validity of comparisons between HC and contemporary study populations.

Although HC approaches have been explored in various therapeutic areas, their application in OA research remains limited. Given the high prevalence and substantial disease burden of OA, it is crucial to assess whether HC methodologies can provide a viable alternative to traditional placebo-controlled RCTs in this field. The present study aims to critically evaluate the feasibility of utilizing a historical placebo arm in OA trials by systematically re-analyzing data from previously published RCTs. Specifically, the objective is to quantify the extent of bias introduced by using HC data and determine whether propensity score matching or other adjustment techniques can effectively align baseline characteristics between historical and contemporary control groups. The study will assess treatment efficacy outcomes in the full trial cohorts and in propensity score-matched subsets to evaluate the impact of different methodological approaches on bias reduction. By examining the comparability of placebo responses across distinct trials, this research will provide insight into the validity and limitations of historical placebo substitution in OA clinical trials.

## 2. Materials and Methods

The objective of this study was to evaluate the feasibility of employing an HC in OA clinical trials. To this end, treatment outcomes from three contemporary RCTs were compared with those from reconstructed historical placebo groups. Specifically, the objective was to assess whether statistical matching techniques could adequately mitigate bias when substituting a real placebo group with an HC arm.

The present study utilized data from three previously published RCTs investigating the efficacy of different treatments for knee OA [11,12,13]. In each of the three original studies, the study protocol was approved by the ethics committee of all participating study centers. All patients provided written informed consent prior to participation. These trials shared some key methodological features, including double-blind randomization, placebo-controlled design, and a primary focus on pain reduction using standardized outcome measures.

1. The first trial, conducted by Kahan et al. in 2009 (RCT2009), was a multicenter, double-blind, placebo-controlled study evaluating the efficacy of a symptomatic slow-acting drug for OA (SYSADOA) over a six-month follow-up period [11]. The study enrolled patients diagnosed with knee OA according to the American College of Rheumatology (ACR) criteria, with moderate to severe pain at baseline. Participants were randomized to receive either the active treatment or a placebo, with pain reduction measured as the primary outcome using the 100 mm visual analogue scale (VAS) at multiple time points.
2. The second trial, conducted by Zegels et al. in 2013 (RCT2013), followed a similar study design, assessing the effects of a pharmacological intervention for knee OA over a shorter, three-month follow-up period [12]. Patients with radiographically confirmed knee OA and persistent pain despite standard symptomatic management were randomized to receive either the experimental treatment or a placebo. The primary outcome measure was pain reduction, evaluated using the 100 mm VAS after three months of treatment.
3. The third study, conducted by Reginster et al. in 2017 (RCT2017), investigated the efficacy of a different treatment for OA symptoms over a six-month follow-up period [13]. The study enrolled patients meeting ACR diagnostic criteria for knee OA who required chronic symptomatic treatment due to persistent pain. Participants were randomly assigned to receive the active treatment or a placebo, and treatment efficacy was assessed primarily through changes in 100 mm VAS pain scores.

In all these studies, pain was analyzed as a continuous outcome and defined as the absolute change in the 100 mm VAS score from baseline to the specified follow-up time points. No binary or responder-based definition of pain improvement was used.

To meet the objective of the present study, the analysis proceeded in three distinct phases:

1. Development of HC Replacement Techniques

To establish an optimal method for generating historical placebo arms, we initially compared the between-groups difference in pain reduction observed in the RCT2013 treated group versus an HC group created from the RCT2009 placebo group and the between-groups real difference observed in the RCT2013 trial. Different statistical techniques were tested to adjust for between-groups differences between study populations, including the following:

- Direct adjustment methods based on observed baseline characteristics;
- Matching techniques, including exact matching and nearest-neighbor approaches;
- Propensity score matching (PSM) to balance covariates between groups.

The goal was to determine whether any of these techniques could successfully align the between-groups difference in pain reduction between the observed one from RCT2013 and the created one by replacing the placebo group by the HC group from RCT2009.

To do so, we first tried to select best baseline characteristics to include as adjustment factors in the direct adjustment method or as matching factors for matching techniques and propensity analyses. Considered baseline characteristics were age, sex, body mass index (BMI), assessed pain, and the duration of OA. To investigate baseline characteristics between the two trials, we proceed in two steps:

- Identify baseline characteristics that differ between RCT2009 and RCT2013 trials. For these comparisons, we summarized baseline characteristics using mean and standard deviations (±SD) or using median and interquartile range (Q1–Q3) when appropriate for quantitative variables and using frequency and percentage for qualitative variables. These descriptive statistics were supplemented by 95% confidence intervals (95%CI). Variables with disjoint 95% confidence intervals indicate some significant difference between the trials and were then considered in the following steps;
- Identify baseline characteristics that show a statistically significant impact on pain reduction in at least one trial. For each trial, we compared pain reduction in the placebo group of the RCT2009 trial and the tested group of the RCT2013 group depending on the participants’ sex using Student’s *t*-test, and we performed Pearson’s or Spearman’s correlation test to evaluate the correlation between pain reduction and participants’ age, BMI, baseline pain, and duration of OA. Significant variables at 95% confidence levels (*p* < 0.05) were then considered in the following steps.

Using the identified baseline characteristics, we tested different techniques to integrate historical placebo data from RCT2009 in RCT2013 data with the objective of correct trials differences.

- The direct adjustments method was conducted by pooling the two databases without any methods of matching the data. The main outcome, pain reduction after 3 months of treatment compared to baseline, was compared between the treatment group (RCT2013) and the placebo group (RCT2009) using a linear model adjusted by baseline characteristics identified in the first step. The between-group mean difference and the associated 95% confidence interval were then estimated using the least squared mean estimates from the linear model;
- The second applied method was matching subjects from the two databases by using the baseline characteristics identified in the first step. In this method, we tried to find a unique matched placebo participant from the RCT2009 trial to each treated participant from the RCT2013 trial based on identified baseline characteristics. Due to the number of treated participants and the number of placebo participants, we performed 1:1 matching; for each treated participant, we assigned one placebo participant, and one placebo participant can be matched to a maximum of one treated participant. In a first approach, we tried to perform an exact matching method, but this approach was too restrictive, and loss of participants was too large. Thus, we preferred a nearest-neighbor approach by authorizing a matching of participants from the two trials within a range of values for quantitative matching characteristics (age: ±2 years, BMI: ±1 kg/m^2^, VAS: ±5 mm, OA duration: ±6 months except for subjects with an OA duration equal to 0 for whom an exact matching was considered). From the matched sample, we estimated the mean of the between-groups difference and its associated 95% confidence interval;
- The propensity score matching method was also considered using the identified baseline characteristics. This method allows to match subjects based on baseline characteristics to control the difference between trials but was less restrictive than the classic matching technique. Due to the number of participants in each trial, we considered 1:1 matching. From the sample obtained using the propensity score, we also estimated the mean of the between-groups difference and its associated 95% confidence interval.

Between-groups differences obtained using HC replacement techniques were then compared with the between-groups difference observed from the RCT2013 trial obtained using an adjusted linear model; the linear model was adjusted by potential confounding factors identified as baseline covariables that potentially impact the reduction of pain as previously.

2. Validation in an Independent RCT

Once a preferred matching technique was identified, it was applied to the RCT2017 trial to test its validity using the same methodology using the same RCT2009 placebo group as the HC group: (1) identify baseline characteristics to include in techniques, (2) apply HC replacement techniques, and (3) compare the between-groups difference compared to the real between-groups difference observed in RCT2017.

3. Post Hoc Analyses and Sensitivity Testing

Since initial attempts to develop an effective HC replacement technique were unsuccessful, additional post hoc analyses were conducted to investigate sources of variability in placebo responses across trials:

- Pairwise placebo comparisons (RCT2013 vs. RCT2009 placebo groups) were performed using Student’s *t*-tests for independent samples and adjusted linear models. Adjustment factors were defined using the same methodology as described above. Cohen’s d effect size for the difference between means was also calculated with its 95% confidence interval;
- As the considered trials present some differences in their baseline characteristics, we also investigated, as sensitivity analyses, if patients with similar profiles between both trials had a similar response to the placebo. We therefore matched patients from the RCT2013 placebo group and the RCT2009 HC group based on identified baseline covariates. An exact matching was not possible without a too large loss of participants. Thus, we defined some range for quantitative characteristics and applied the nearest-neighbors approach. Propensity score matching was also performed. With these matched samples, we compared the pain reduction between the two placebo groups using 95% confidence intervals of the between-groups difference.

The same approaches were used to compared the RCT2017 placebo group to the RCT2009 HC.

## 3. Results

### 3.1. Development of HC Replacement Techniques

To develop HC replacement techniques, we first compared baseline characteristics between the RCT2009 HC group and the RCT2013 treated group (Table 1). Based on 95% confidence intervals, we observed that RCT2013 participants were slightly older and had higher pain at baseline than RCT2009 participants. In both trials, a significant absolute reduction of pain between 3 months of treatment and baseline was observed (Table 1); this reduction of pain was correlated with pain observed at baseline (*p* < 0.0001). Other baseline characteristics did not significantly impact the reduction of pain.

**Figure 1:**
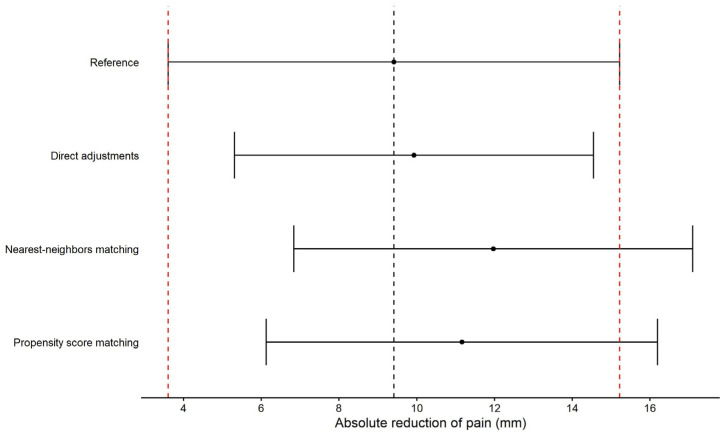
Comparisons of HC replacement techniques for RCT2013 trial; points and error bars represent the between-groups mean difference in reduction of pain with the associated 95% confidence intervals; black dashed line represents the between-groups difference observed in RCT2013 trial and the red dashed lines represent the 95% confidence interval limits.

Based on previous results, age and pain at baseline were used as adjustments for the direct adjustments method and as matching factors for the nearest-neighbors and propensity score matching methods. The reference between-groups mean difference observed in the RCT2013 trial as well as the estimated between-groups mean difference from each of the HC replacement methods are displayed with their 95% confidence intervals in Figure 1. This figure presents the estimated mean between-group differences accompanied by their 95% confidence intervals. The ‘reference’ corresponds to the estimated mean derived from RCT2013 and serves as the reference for assessing whether HC replacement techniques can correctly estimate the difference between the treated and placebo groups. The estimated mean between-group differences for each technique are then displayed, together with their corresponding 95% confidence intervals. Based on this plot, we observe that, overall, the three methods tend to overestimate the between-group difference, as the estimated means are higher than the reference. However, the confidence intervals overlap with that of the reference, indicating that the between-group differences are comparable between the reference and the replacement techniques.

### 3.2. Validation in an Independent RCT

Using the encouraging results of each HC replacement method developed with RCT2013, the same methods were performed to create an HC group from RCT2009 for the RCT2017 trial. Participants in the RCT2017 treated group were older and had higher pain at baseline than in the RCT2009 placebo group. Moreover, the proportions of women were also higher in the RCT2017 treated group than in the RCT2009 placebo group (Table 1). These factors were then considered as adjustments factors. As, in the RCT2017 treated group, the duration of OA was significantly correlated with the reduction of pain, duration of OA was also included as an adjustment factor.

**Figure 2:**
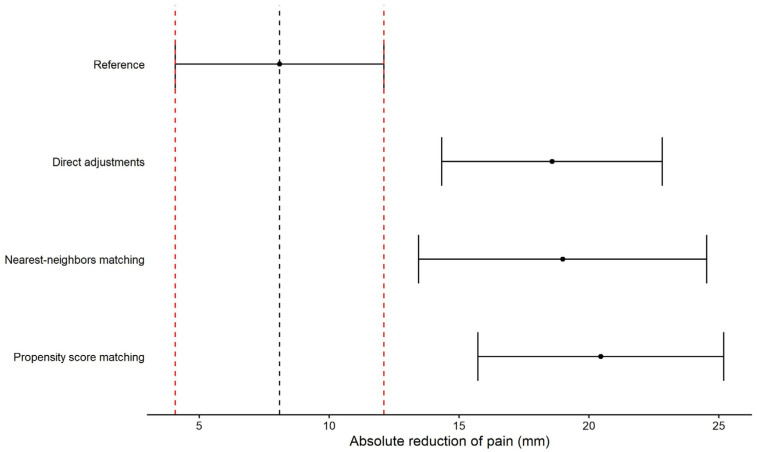
Comparisons of HC replacement techniques for RCT2017 trial; points and error bars represent the between-groups mean difference in reduction of pain with the associated 95% confidence intervals; black dashed line represents the between-groups difference observed in RCT2017 trial and the red dashed lines represent the 95% confidence interval limits.

Using these adjustments factors, between-groups differences estimated using each of the HC replacement methods were compared to that of the reference obtained by the RCT2017 trial (Figure 2). Despite the adjustments factors to take account of the difference between each trial characteristic, each of the HC replacement methods overestimated the between-groups differences.

The use of RCT2013 as an HC group for the treated group from RCT2017 can also be considered to provide a second validation of the different HC replacement techniques. This analysis, provided in detail in Supplementary File S1, tends to confirm the results presented in the article. In this analysis, despite improved comparability of socio-demographic characteristics, the HC replacement techniques did not yield convincing results.

### 3.3. Post Hoc Analyses and Sensitivity Testing

To explain the results obtained by HC replacement methods in both the RCT2013 and RCT2017 trials by using the RCT2009 HC group, we compared the reduction of pain in the placebo groups from each trial (Table 2).

Concerning baseline characteristics, we observed that, in the RCT2013 placebo group, participants were older and had higher pain at baseline than participants from the RCT2009 HC group. Participants of the RCT2017 placebo group were older, had a higher BMI, and had higher pain at baseline than participants from the RCT2009 HC group.

The absolute reduction of pain at 3 months compared to baseline in RCT2013 was comparable to the absolute reduction of pain in RCT2009 (*p* > 0.05) with a small effect size equal to *d* = 0.13. By taking account of the difference in characteristics at baseline, we also observed a mean between-groups difference which did not significantly differ from zero (Table 3). As sensitivity analyses, we also compared the between-groups difference in a subset of participants with similar characteristics in both trials. Using matching or propensity score, the pain reduction in each group was comparable as observed previously (Table 3).

In contrast, the absolute reduction of pain at 6 months compared to baseline in RCT2017 was significantly greater than in RCT2009 (*p* < 0.05). The effect size of the between-group difference indicated a medium effect size (*d* = 0.67) between the reduction of pain in the RCT2017 placebo group and in the RCT2009 HC group. By taking account of difference in baseline characteristics, the adjusted mean between-groups difference also indicated a difference in absolute pain reduction in the placebo groups between both trials (Table 4). Sensitivity analyses performed on a subset of matched participants with similar characteristics from the two trials also showed a significant difference in the reduction of pain between the placebo groups of the two trials (Table 4).

## 4. Discussion

The present study sought to evaluate the feasibility of substituting a contemporary placebo control group with a historical placebo arm in OA clinical trials by applying various statistical matching techniques. An important observation was the significant discrepancy in placebo responses between trials, despite the application of multiple adjustment techniques, including direct covariate adjustment, matching methods, and propensity score approaches. Initial efforts to develop a matching strategy using data from RCT2009 and RCT2013 yielded encouraging results; however, validation in the RCT2017 trial revealed that historical placebo responses continued to differ substantially from those observed in a real-time placebo arm. This discrepancy remained evident even after accounting for key confounding variables such as age, sex, BMI, and baseline pain scores. The findings of the study highlight significant challenges associated with these matching techniques, revealing substantial variability in placebo responses across different trials despite attempts to match baseline characteristics. The study’s findings suggest that historical placebo substitution may not be a viable alternative in OA RCTs due to persistent methodological and statistical limitations.

The inability to adequately align placebo responses across trials can be attributed to multiple factors. Firstly, unmeasured confounders may have played a critical role in influencing placebo effects [14,15]. While the matching approaches that were employed took into account major demographic and clinical variables, other patient-specific characteristics, such as prior NSAID use, concomitant therapies, disease severity beyond pain scores, and psychological factors (e.g., patient expectations), were not captured in the available datasets. The presence of these unmeasured variables may have resulted in differential placebo responses across trials [16]. Secondly, placebo outcomes may have been influenced by differences in study design and execution [17,18]. Although the trials appeared broadly comparable, they differed in several methodological and contextual elements that may influence placebo and active treatment responses. In particular, the Kahan et al. trial was conducted between 2000 and 2004, almost a decade before the two other studies, and pain was not the primary endpoint. Furthermore, this study had a two-year total duration, whereas the other trials assessed pain over three or six months. Variations in patient recruitment strategies, and regional or site-specific factors, could also have introduced heterogeneity in placebo effects. Placebo response is known to be influenced by patient–clinician interactions, cultural expectations, and differences in standard of care across study sites, all of which could contribute to discrepancies between historical and contemporary placebo groups. Thirdly, statistical limitations inherent to the use of HC data must be considered. Propensity score matching and other statistical adjustments assume that all relevant confounders can be accounted for through observed covariates. However, in the absence of truly randomized allocation between historical and contemporary cohorts, concerns regarding residual confounding persist [19]. Furthermore, propensity score techniques are only able to perform optimally when there is substantial overlap in baseline characteristics between groups [20]. In the present study, despite efforts to align patient profiles across trials, the observed placebo response variability suggests that true comparability was not achieved.

The challenges identified in this study are consistent with those previously identified in other research on the limitations of HC designs in clinical trials. Prior studies in other therapeutic areas have demonstrated that historical placebo responses can be highly variable and influenced by factors beyond those captured in routine clinical datasets. For instance, meta-analyses of placebo responses in chronic pain conditions have shown that placebo effects can change over time due to evolving patient expectations, improved standards of care, and changes in trial methodologies [21,22]. A similar phenomenon has been observed in OA research, where placebo responses have been shown to change over time, potentially due to heightened awareness of the placebo effect and advancements in symptomatic management [23].

While some studies have successfully employed HC data in the development of pharmaceuticals, these successes have been primarily observed in diseases with well-characterized, stable natural histories and highly predictable disease progression (e.g., rare genetic disorders) [24]. In contrast, OA is a heterogeneous condition influenced by multiple individual, environmental, and treatment-related factors, making the substitution of real-time placebo groups particularly challenging. The present findings serve to reinforce the notion that HC approaches should be applied with the utmost caution, particularly in conditions where placebo response is highly variable.

This study has both strengths and limitations. A key strength of this study is its utilization of data from well-conducted, double-blind, placebo-controlled RCTs with similar patient populations and outcome measures. The rigorous statistical approaches applied, including multiple matching and adjustment methods, allowed for a comprehensive assessment of the feasibility of historical placebo substitution. However, it is important to acknowledge several limitations. Firstly, while the included studies were methodologically similar, they were not identical in terms of study duration, patient demographics, and treatment regimens. These variations may have contributed to the observed variations in placebo responses. Secondly, unmeasured confounding factors, including psychosocial variables and treatment expectations, could not be accounted for in the matching process. An additional limitation of this study relates to some variability in pain intensity and pain trajectories across the included RCTs. Although baseline pain was explicitly accounted for in adjustment and matching procedures, differences in pain experience over time, influenced by trial-specific factors such as study design, follow-up duration, and contextual effects, may not be fully captured by statistical models. Such variability may have contributed to heterogeneous placebo responses and further limits the generalizability of historical placebo substitution in OA trials. Finally, the selection of historical placebo cohorts was partly opportunity-driven, reflecting access to individual-level data from previously conducted RCTs. While the included studies shared several key methodological features, this pragmatic selection approach may limit generalizability. However, it also reflects real-world constraints commonly encountered when attempting to use historical controls and reinforces the relevance of the observed lack of reproducibility.

The present findings emphasize the necessity for prudence when contemplating the utilization of historical placebo controls in OA research. While HC approaches may offer certain advantages in terms of reducing the necessity for new placebo-controlled arms, their application in OA RCTs remains highly challenging due to the unpredictability of placebo responses. It is imperative that future research endeavors concentrate on delineating the circumstances under which historical placebo substitution could be a viable option. This could include (1) greater harmonization of study protocols, including patient selection criteria, trial duration, and outcome assessment methods, to improve comparability between trials; (2) exploring alternative statistical methods such as machine learning techniques and Bayesian hierarchical models that may provide more sophisticated approaches to adjusting for inter-trial variability in placebo responses; and (3) the identification of individual-level factors that contribute to placebo variability, such as psychological and behavioral characteristics, that could enhance the accuracy of matching techniques.

## 5. Conclusions

In conclusion, the present study indicates that the substitution of a contemporaneous placebo group with historical placebo data is not currently applicable in OA RCTs. Despite extensive adjustment and matching strategies, substantial and non-reproducible differences in placebo responses persisted across trials. These findings suggest that the high variability of placebo effects in OA, combined with heterogeneity in study design and conduct, limits the validity of historical placebo approaches in this field. From a broader perspective, historical placebo substitution may only be feasible under highly restrictive conditions, such as the availability of near-identical (“twin”) trials with harmonized inclusion criteria, outcome definitions, follow-up duration, and study conduct. Additional requirements would include comprehensive capture of individual-level factors known to influence placebo response, as well as the use of advanced analytical frameworks capable of modeling inter-trial heterogeneity. Until such conditions are met, historical placebo controls should be used with extreme caution in OA research.

## Figures and Tables

**Table 1:** Study characteristics.

	RCT2009 HC Group	RCT2013 Treated Group	RCT2017 Treated Group
**Study design**	Multicenter, double-blind, placebo-controlled study	Multicenter, double-blind, placebo-controlled study	Multicenter, double-blind, placebo-controlled study
**Study objective**	Evaluate the efficacy of a symptomatic slow-acting drug for OA (SYSADOA) over a six-month follow-up period	Assess the effects of a pharmacological intervention for knee OA over a three-month follow-up period	Investigate the efficacy of a different treatment for OA symptoms with follow-ups at both three and six months
**Study population**	Patients diagnosed with knee OA according to ACR criteria, with moderate to severe pain at baseline	Patients with radiographically confirmed knee OA and persistent pain despite standard symptomatic management	Patients meeting ACR diagnostic criteria for knee OA who required chronic symptomatic treatment due to persistent pain
**Study primary endpoint**	Pain reduction using the 100 mm VAS at multiple time points included three and six months of treatment	Pain reduction evaluated using the 100 mm VAS after three months of treatment	Changes in 100 mm VAS pain scores at both three and six months
**Baseline characteristics**	*N* = 313	*N* = 236	*N* = 333
Age (years)—mean ± SD (95%CI)	61.75 ± 8.50 (60.81–62.70)	65.33 ± 9.60 (64.10–66.57)	65.33 ± 7.67 (64.50–66.16)
Sex—female proportion (95%CI)	66.8% (61.3–72.0%)	63.1% (56.6–69.3%)	79.6% (75.3–83.9%)
BMI (kg/m^2^)—mean ± SD (95%CI)	28.96 ± 5.24 (28.37–29.54)	28.58 ± 4.81 (27.96–29.20)	29.77 ± 4.51 (29.29–30.26)
Duration of OA (year)—median [Q1–Q3] (95%CI)	4.0 [2.0–10.0] (4.0–5.0)	5.0 [2.0–11.0] (5.0–7.0)	3.8 [1.7–7.2] (3.1–4.6)
Pain at baseline (mm)—mean ± SD (95%CI)	57.24 ± 17.31 (55.32–59.17)	63.97 ± 14.32 (62.13–65.80)	69.81 ± 9.68 (68.76–70.85)
**Pain assessed at 3 months**	*N* = 294	*N* = 206	
Pain at 3 months (mm)—mean ± SD (95%CI)	43.14 ± 27.32 (40.00–46.27)	35.77 ± 23.69 (32.51–39.02)	
Absolute reduction of pain (mm)—mean ± SD (95%CI)	−14.11 ± 26.21 (−17.13–−11.10)	−27.65 ± 26.69 (−31.32–−23.98)	
**Association of pain reduction at 3 months with baseline characteristics**			
Age (years)	$r_{P}$ = −0.11, *p* = 0.060	$r_{P}$ = −0.09, *p* = 0.22	
Sex—mean ± SD	*p* = 0.17	*p* = 0.89	
Men	−17.17 ± 25.46	−27.99 ± 25.87	
Women	−12.65 ± 26.50	−27.44 ± 27.28	
BMI (kg/m^2^)	$r_{P}$ = −0.03, *p* = 0.61	$r_{P}$ = −0.08, *p* = 0.27	
Duration of OA (weeks)	$r_{S}$ = −0.11, *p* = 0.073	$r_{S}$ = −0.11, *p* = 0.12	
Pain at baseline (mm)	$r_{P}$ = −0.27, *p* < 0.001	$r_{P}$ = −0.47, *p* < 0.001	
**Pain assessed at 6 months**	*N* = 282		*N* = 333
Pain at 6 months (mm)—mean ± SD (95%CI)	39.67 ± 27.79 (36.42–42.93)		27.33 ± 21.60 (25.00–29.66)
Absolute reduction of pain (mm)—mean ± SD (95%CI)	−17.18 ± 26.95 (−20.34–−14.02)		−42.48 ± 22.53 (−44.91–−40.05)
**Association of pain reduction at 6 months with baseline characteristics**			
Age (years)	$r_{P}$ = −0.069, *p* = 0.25		$r_{P}$ = 0.0002, *p* = 0.99
Sex—mean ± SD	*p* = 0.17		*p* = 0.099
Men	−20.25 ± 24.95		−46.81 ± 21.28
Women	−15.71 ± 27.79		−41.37 ± 22.75
BMI (kg/m^2^)	$r_{P}$ = 0.020, *p* = 0.73		$r_{P}$ = −0.010, *p* = 0.86
Duration of OA (weeks)	$r_{S}$ = −0.048, *p* = 0.43		$r_{S}$ = 0.16, *p* = 0.0029
Pain at baseline (mm)	$r_{P}$ = −0.28, *p* < 0.001		$r_{P}$ = −0.31, *p* < 0.001

Mean ± SD: mean and standard deviation; median [Q1–Q3]: median and interquartile range; 95%CI: 95% confidence interval; $r_{P}$: Pearson’s correlation coefficient; $r_{S}$: Spearman’s correlation coefficient.

**Table 2:** Placebo groups baseline characteristics.

	RCT2009 HC Group	RCT2013 Placebo Group	RCT2017 Placebo Group
**Baseline characteristics**	*N* = 313	*N* = 117	*N* = 205
Age (years)—mean ± SD (95%CI)	61.75 ± 8.50 (60.81–62.70)	64.91 ± 10.56 (62.98–66.85)	64.94 ± 8.04 (63.83–66.04)
Sex—female proportion (95%CI)	66.8% (61.3–72.0%)	67.5% (58.2–75.9%)	74.2% (67.6–80.0%)
BMI (kg/m^2^)—mean ± SD (95%CI)	28.96 ± 5.24 (28.37–29.54)	28.62 ± 6.10 (27.51–29.74)	30.60 ± 4.99 (29.91–31.29)
Duration of OA (weeks)—median [Q1–Q3] (95%CI)	4.0 [2.0–10.0] (4.0–5.0)	7.0 [2.0–11.5] (5.0–9.0)	4.2 [1.8–6.9] (3.7–4.8)
Pain at baseline (mm)—mean ± SD (95%CI)	57.24 ± 17.31 (55.32–59.17)	62.54 ± 14.98 (59.79–65.28)	69.99 ± 10.36 (68.56–71.42)

Mean ± SD: mean and standard deviation; median [Q1–Q3]: median and interquartile range; 95%CI: 95% confidence interval.

**Table 3:** Pain characteristics in placebo groups from RCT2009 and RCT2013 at 3 months.

	RCT2009 HC Group	RCT2013 Placebo Group
**Pain assessed at 3 months**	*N* = 294	*N* = 97
Pain at 3 months (mm)—mean ± SD (95%CI)	43.14 ± 27.32 (40.00–46.27)	44.65 ± 25.18 (39.57–49.72)
Absolute reduction of pain (mm)—mean ± SD (95%CI)	−14.11 ± 26.21 (−17.13–−11.10)	−17.48 ± 25.62 (−22.65–−12.32)
**Between-groups difference—mean (95%CI); Cohen’s *d***	−3.37 (−9.37; 2.63); *d* = 0.13 (−0.10; 0.36)	
**Adjusted between-groups difference—mean (95%CI)**	−0.34 (−6.19; 5.51)	
**Matching between-groups difference—mean (95%CI)**	−7.14 (−20.45; 6.16) − *N* = 28 matched pairs	
**Propensity between-groups difference—mean (95%CI)**	−1.07 (−8.63; 6.50) − *N* = 91 matched pairs	

Mean ± SD: mean and standard deviation; 95%CI: 95% confidence interval.

**Table 4:** Pain characteristics in placebo groups from RCT2009 and RCT2017 at 6 months.

	RCT2009 HC Group	RCT2017 Placebo Group
**Pain assessed at 6 months**	*N* = 282	*N* = 172
Pain at 3 months (mm)—mean ± SD (95%CI)	39.67 ± 27.79 (36.42–42.93)	35.57 ± 23.46 (32.04–39.10)
Absolute reduction of pain (mm)—mean ± SD (95%CI)	−17.18 ± 26.95 (−20.34–−14.02)	−34.62 ± 24.17 (−38.25–−30.98)
**Between-groups difference—mean (95%CI); Cohen’s *d***	−17.44 (−22.37; −12.51)—*d* = 0.67 (0.48–0.87)	
**Adjusted between-groups difference—mean (95%CI)**	−11.92 (−17.41; −6.69)	
**Matching between-groups difference—mean (95%CI)**	−12.22 (−20.99; −3.44) − *N* = 66 matched pairs	
**Propensity between-groups difference—mean (95%CI)**	−14.66 (−29.19; −10.68) − *N* = 146 matched pairs	

Mean ± SD: mean and standard deviation; 95%CI: 95% confidence interval.

## Data Availability

The datasets presented in this article are not readily available because they are part of clinical trials owned by IBSA. Requests to access the datasets should be directed to the corresponding author. The current use of these datasets is consistent with the original consent/approvals and applicable data governance.

## Supplementary Materials

The following supporting information can be downloaded at: https://www.mdpi.com/article/10.3390/epidemiologia7010027/s1, File S1: Supplementary file to the manuscript “Evaluating the feasibility of using historical placebo control in osteoarthritis trials”.

## Abbreviations

The following abbreviations are used in this manuscript:

ACR	American College of Rheumatology
BMI	body mass index
HC	historical control
OA	osteoarthritis
PSM	propensity score matching
RCT	randomized controlled trial
SD	standard deviation
SYSADOA	symptomatic slow-acting drug for osteoarthritis
VAS	visual analogue scale
