# Supplementary material for: Evaluating the Feasibility of Using Historical Placebo Control in Osteoarthritis Trials
Source: Epidemiologia (Basel). 2026 Feb 14;7(1):27. doi: 10.3390/epidemiologia7010027 (PMC12922152; doi:10.3390/epidemiologia7010027)
Supplement: Supplementary file 1 [file epidemiologia-07-00027-s001.zip › epidemiologia-4023651-supplementary.pdf]

Supplementary file to the manuscript "Evaluating the feasibility of using historical placebo control in osteoarthritis trials"

The use of RCT2013 as an HC group for the treated group from RCT2017 can also be considered to provide a second validation of the different HC replacement techniques. The following table presents the characteristics of both groups (RCT2013 HC group and RCT2017 treated group).

	**RCT2013 HC group**	**RCT2017 treated group**
**Baseline characteristics**	N = 117	N = 361
Age (years)—mean ± SD (95%CI)	64.91 ± 10.56 (62.98–66.85)	65.31 ± 7.78 (64.50–66.16)
Sex—female proportion (95%CI)	67.5% (59.0%–76.0%)	80.1% (75.9%–84.2%)
BMI (kg/m^2)—mean ± SD (95%CI)	28.62 ± 6.10 (27.51–29.74)	29.80 ± 4.45 (29.34–30.26)
Duration of OA (weeks)—median [Q1–Q3] (95%CI)	7.0 [2.0–11.5] (5.0–9.0)	3.8 [1.7–7.2] (3.1–4.6)
Pain at baseline (mm)—mean ± SD (95%CI)	62.54 ± 14.98 (56.00–65.00)	70.01 ± 9.66 (69.01–71.01)
**Pain assessed at 3 months**	N = 97	N = 361
Pain at 3 months (mm)—mean ± SD (95%CI)	44.65 ± 25.18 (39.57–49.72)	37.65 ± 22.62 (35.32–40.00)
Absolute reduction of pain (mm)—mean ± SD (95%CI)	-17.48 ± 25.62 (-22.65–-12.32)	-32.34 ± 22.18 (-34.64–-30.05)
**Association of pain reduction at 3 months with baseline characteristics**		
Age (years)	r_P = -0.07, p = 0.48	r_P = 0.02, p = 0.65
Sex—mean ± SD	p = 0.88	p = 0.16
Men	-18.10 ± 22.59	-35.60 ± 22.66
Women	-17.21 ± 27.02	-31.53 ± 22.03
BMI (kg/m^2)	r_P = 0.04, p = 0.69	r_P = 0.03, p = 0.55
Duration of OA (weeks)	r_S = 0.11, p = 0.30	r_S = 0.20, p = 0.0001
Pain at baseline (mm)	r_P = -0.32, p = 0.0014	r_P = -0.17, p = 0.0010

Based on this table, we observed that the socio-demographic characteristics (age, sex, and BMI) were similar between the two RCTs. However, significant differences were present regarding baseline pain and OA duration. The analysis of the association between these characteristics and the absolute reduction of pain at 3 months from baseline highlighted these same two variables. Therefore, the adjustment and matching techniques for HC replacement were performed considering pain at baseline and the duration of OA.

Despite comparability of socio-demographic factors, a set of 75 matched pairs was obtained for nearest-neighbors matching and a set of 90 matched pairs was obtained for propensity score matching. The following figure displays the true between-groups difference (RCT2017 observed between-groups difference) with its 95% confidence interval as well as between-groups differences obtained using the HC replacement techniques.

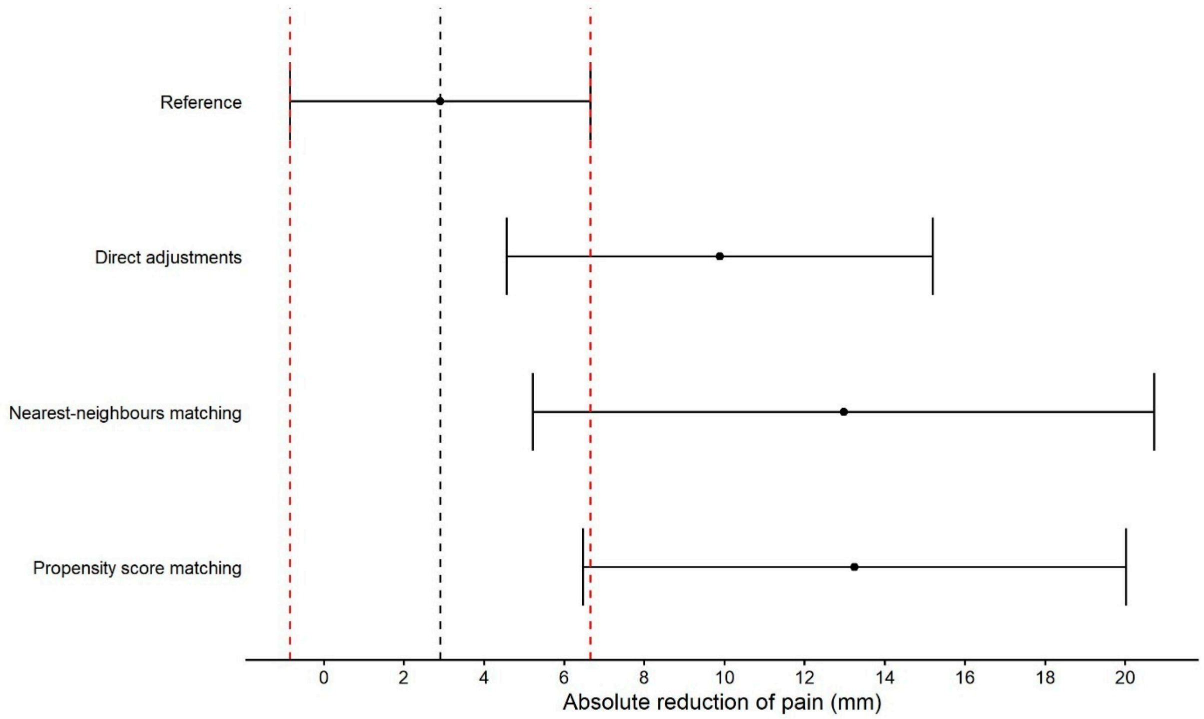

Based on this figure, we observed that, although the 95% confidence interval of the between-groups difference did not allow rejection of the equality, the HC replacement techniques did not lead to convincing results. Indeed, when considering the absolute reduction of pain from baseline to the 3-month results from RCT2017, we cannot reject a difference between the placebo and the treatment. However, when using HC replacement techniques, this leaded to a statistically significant difference in absolute pain reduction from baseline between the treated group and the HC group. Moreover, each HC replacement technique overestimated the between-groups difference observed in RCT2017.

This second analysis therefore tends to confirm the results presented in the article. In this second analysis, despite improved comparability of socio-demographic characteristics, the HC replacement techniques did not yield convincing results.
